# Deep-learning model for evaluating histopathology of acute renal tubular injury

**DOI:** 10.1038/s41598-024-58506-9

**Published:** 2024-04-19

**Authors:** Thi Thuy Uyen Nguyen, Anh-Tien Nguyen, Hyeongwan Kim, Yu Jin Jung, Woong Park, Kyoung Min Kim, Ilwoo Park, Won Kim

**Affiliations:** 1 Department of Histology, Embryology, Pathology and Forensic Medicine, Hue University of Medicine and Pharmacy, Hue University, Hue City, Vietnam; 2 Department of Radiology, Chonnam National University and Hospital, Gwangju, Korea; 3 Department of Medical Informatics, University Medical Center Göttingen, Göttingen, Germany; 4 Department of Internal Medicine, Jeonbuk National University Medical School, Jeonju, Republic of Korea; 5 Research Institute of Clinical Medicine of Jeonbuk National University-Biomedical Research Institute, Jeonju, Republic of Korea; 6 Department of Pathology, Jeonbuk National University Medical School, Jeonju, Korea; 7 Department of Artificial Intelligence Convergence, Chonnam National University, Gwangju, Korea; 8 Department of Data Science, Chonnam National University, Gwangju, Korea

**Keywords:** Deep learning, Acute renal tubular injury, Segmentation, Computational biology and bioinformatics, Nephrology

## Abstract

Tubular injury is the most common cause of acute kidney injury. Histopathological diagnosis may help distinguish between the different types of acute kidney injury and aid in treatment. To date, a limited number of study has used deep-learning models to assist in the histopathological diagnosis of acute kidney injury. This study aimed to perform histopathological segmentation to identify the four structures of acute renal tubular injury using deep-learning models. A segmentation model was used to classify tubule-specific injuries following cisplatin treatment. A total of 45 whole-slide images with 400 generated patches were used in the segmentation model, and 27,478 annotations were created for four classes: glomerulus, healthy tubules, necrotic tubules, and tubules with casts. A segmentation model was developed using the DeepLabV3 architecture with a MobileNetv3-Large backbone to accurately identify the four histopathological structures associated with acute renal tubular injury in PAS-stained mouse samples. In the segmentation model for four structures, the highest Intersection over Union and the Dice coefficient were obtained for the segmentation of the “glomerulus” class, followed by “necrotic tubules,” “healthy tubules,” and “tubules with cast” classes. The overall performance of the segmentation algorithm for all classes in the test set included an Intersection over Union of 0.7968 and a Dice coefficient of 0.8772. The Dice scores for the glomerulus, healthy tubules, necrotic tubules, and tubules with cast are 91.78 ± 11.09, 87.37 ± 4.02, 88.08 ± 6.83, and 83.64 ± 20.39%, respectively. The utilization of deep learning in a predictive model has demonstrated promising performance in accurately identifying the degree of injured renal tubules. These results may provide new opportunities for the application of the proposed methods to evaluate renal pathology more effectively.

## Introduction

Acute kidney injury (AKI) is characterized by sudden decrease in renal function. Pathologists use acute tubular injury (ATI) to describe the histopathological findings of AKI caused by damage to the tubules due to ischemia or toxin-induced toxicity. In practice, rather than using the term acute tubular necrosis (ATN), which has been traditionally employed despite the lack of necrosis in several cases, semiquantitative histopathological assessment of ATI is classified into three levels: mild, moderate, or severe^1^. Although the histopathology of ATI may differ between distinct pathologies, it is generally characterized by focal or diffuse tubular dilatation, thinning of the lining epithelium, vacuolation, loss of the brush border in proximal tubules, loss of nuclei, rupture of the basement membrane, and tubular cast formation in toxic acute tubular injury^2,3^. Kidney Disease: Improving Global Outcomes (KDIGO) urges the discovery of the etiology of AKI whenever possible^4,5^. Histopathological assessment may help distinguish different types of AKI and aid in patient care^1^.

Deep learning is the most recent machine-learning innovation and provides an unrivaled capacity to efficiently manage patients, render diagnostic support, and guide therapies^6–8^. Recent breakthroughs in deep learning, particularly convolutional neural networks (CNNs), have provided new techniques for developing systems that can assist pathologists in clinical diagnoses. Advances in whole-slide imaging technology have promoted new deep learning applications in renal histopathology^9,10^. Pathologists' common tasks of recognizing and identifying tissue components can be decomposed into computer vision tasks such as segmentation and detection.

Various deep learning algorithms have recently been developed for the multiclass segmentation of whole renal slide images from human and mouse kidney diseases. Most studies have focused on glomerular segmentation^11^. Recently, Massimo Salvi et al.^12^ demonstrated that an automated method using the RENTAG algorithm may be effective in quantifying glomerulosclerosis and tubular atrophy. However, few studies have used deep-learning models for the histopathological assessment of renal tubular injury after AKI. Therefore, this study was conducted to apply deep-learning models to the histopathological segmentation of the four structures in acute renal tubular injury.

In summary, our contributions are as follows. A segmentation model was developed using the DeepLabV3 architecture to accurately identify the four histopathological structures associated with acute renal tubular injury: glomerulus, necrotic tubules, healthy tubules, and tubules with cast. Our approach achieves promising performance in accurately identifying the degree of injured renal tubules.

## Material and method

### Kidney sample and criteria of acute kidney injury

This study was performed with the approval of the Ethical Committee of Jeonbuk National University Hospital. All methods were performed in accordance with the relevant guidelines and regulations. In a previous study, kidney samples were collected from a mouse model of cisplatin-induced acute tubular injury^13^. We re-analyzed kidney samples from male C57BL/6 mice (age: 8–9 weeks; weight: 20–25 g). The mice were divided into two groups: control buffer-treated and cisplatin-treated. Mice in the cisplatin group were intraperitoneally administered a single dose of cisplatin (Cis; 20 mg/kg; Sigma Chemical Co., St. Louis, MO, USA), whereas mice in the control group were intraperitoneally administered saline. Histological measurements were performed 72 h after treatment with cisplatin or the control buffer. To evaluate the function of the injured kidney, blood samples were collected three days after cisplatin administration to measure serum creatinine levels. When serum creatinine was above 0.5 mg/dL, acute kidney injury caused by cisplatin was determined.

### Histopathology and assessment of tubular injury

Kidney tissue was fixed in formalin and embedded in paraffin blocks. Hematoxylin and eosin (HE) staining was performed to assess renal tubular injury. Sections of 3-µm thickness were stained using the Periodic acid-Schiff (PAS) Stain Kit (Abcam, Cambridge, MA, USA; catalog no. 150680) in accordance with the manufacturer’s instructions^12,14^. Tubular injury was evaluated by three blinded observers who examined at least 20 cortical fields (× 200 magnification) of the PAS-stained kidney sections. Tubular injury (necrotic tubules) was defined as tubular dilation, tubular atrophy, tubular cast formation, brush border loss, or thickening of the tubular basement membrane. Finally, the slides were digitized using a Motic Easy ScanPRO slide scanner (Motic Asia Corp., Kowloon, Hong Kong) at 40× magnification.

### Datasets

**Figure 1:**
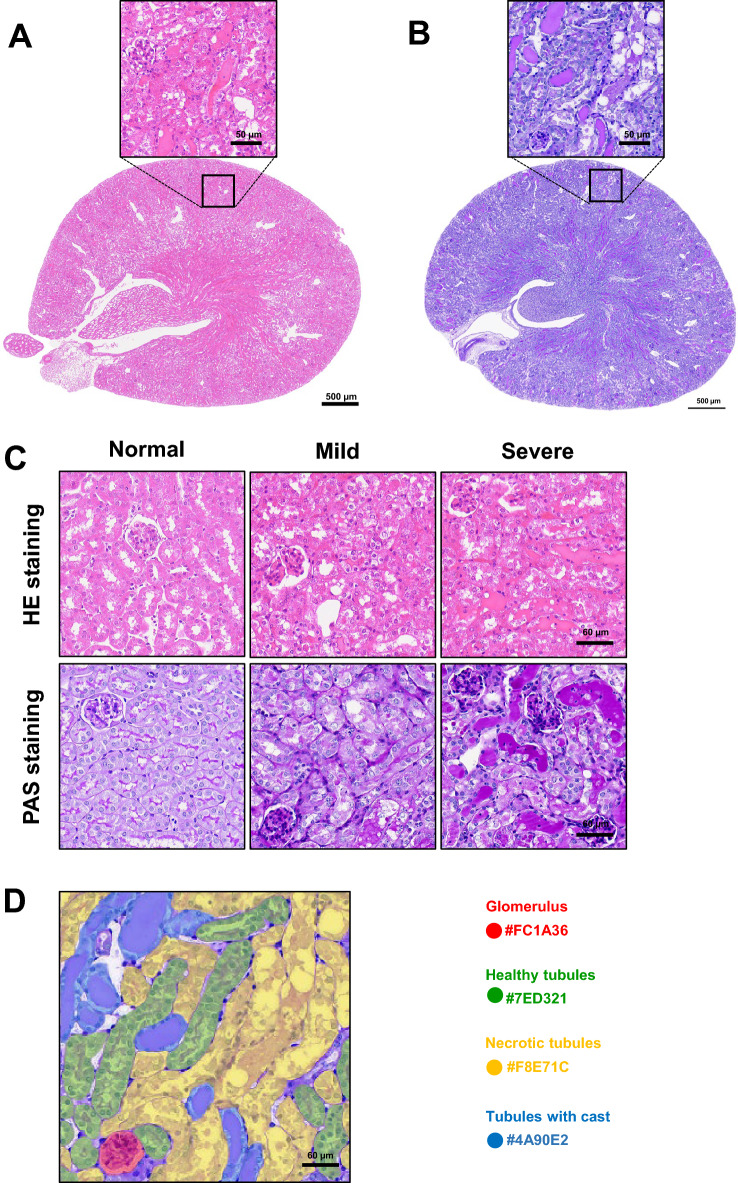
**A**–**B** Whole slide image of H & E (**A**) and PAS (**B**)-stained kidney section was digitalized using slide scanner at 40× magnification. Randomly generated patch without annotations. **B** H&E and PAS staining images of healthy tubules, necrotic tubules, and tubules with casts after cisplatin administration. **C** Randomly generated patch with annotations comprised four different structures: “glomerulus,” “healthy tubules,” “necrotic tubules,” and “tubule with cast”.

**Figure 2:**
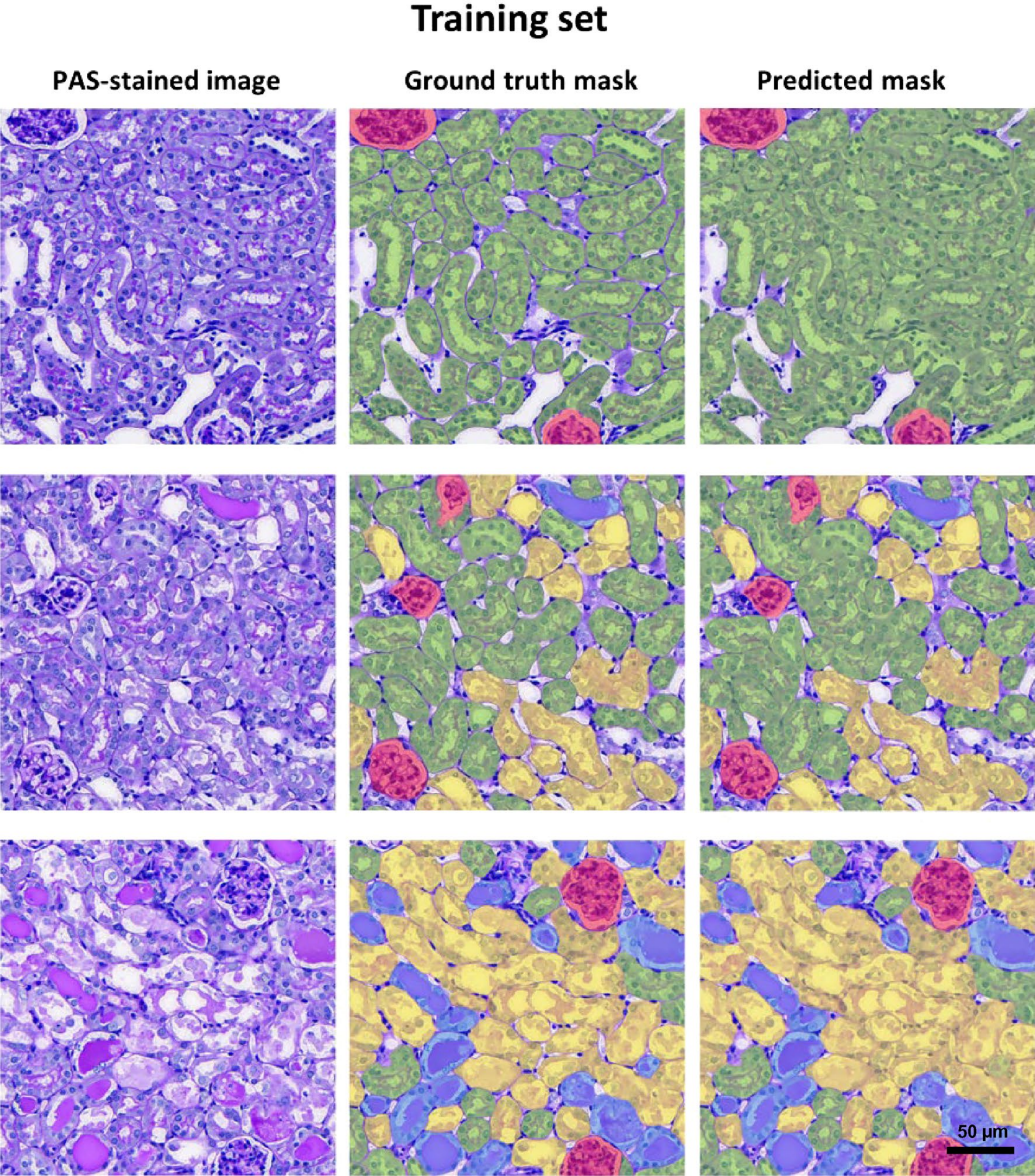
Representative PAS-stained images, ground truth mask and predicted mask generated by the CNNs in training set.

**Figure 3:**
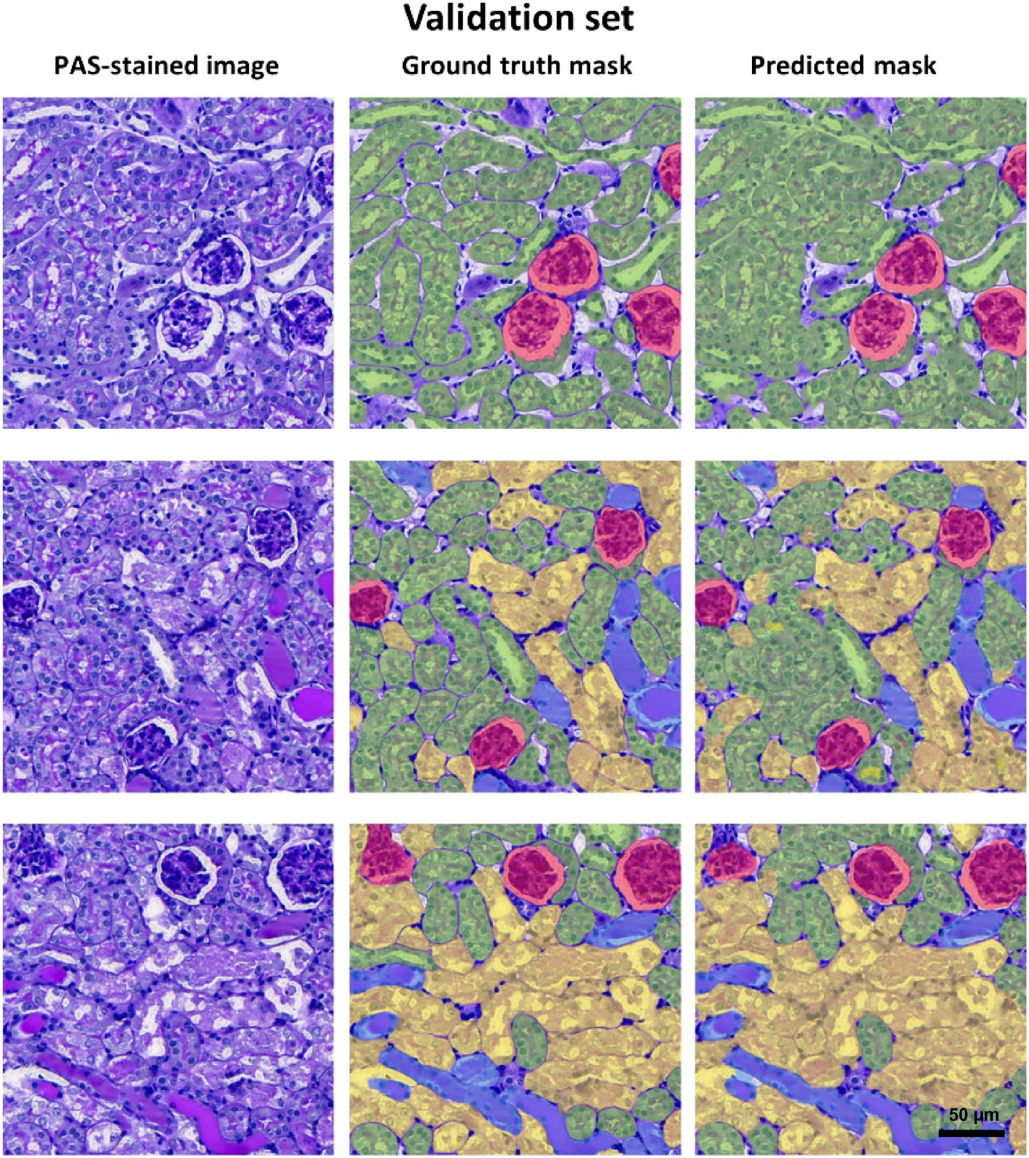
Representative PAS-stained images, ground truth mask and predicted mask generated by the CNNs in validation set.

**Figure 4:**
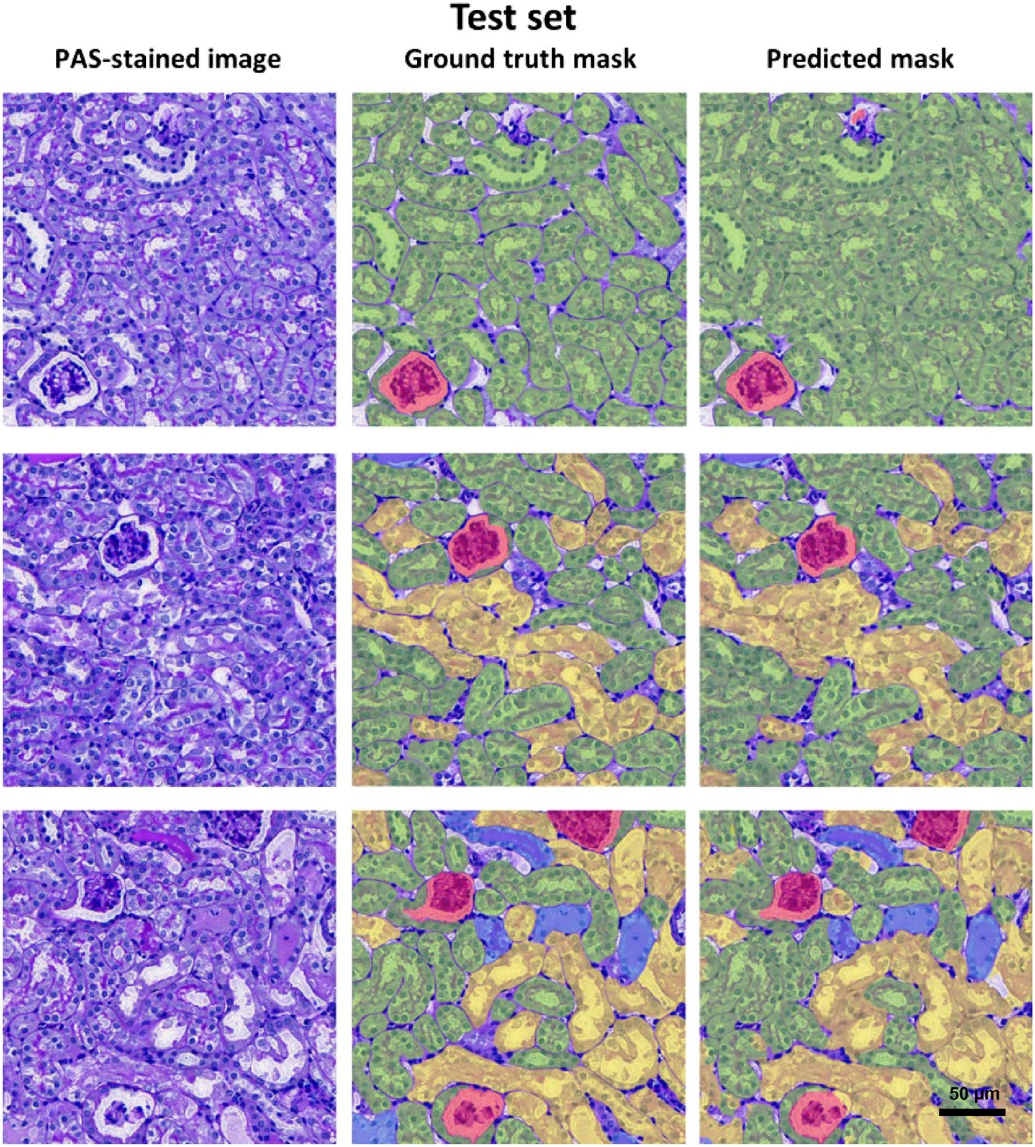
Representative PAS-stained images, ground-truth masks, and predicted masks generated by CNNs in test set.

Forty-five whole-slice images (WSIs) with 400 generated patches were used for the segmentation model devolopment. Ground-truth annotations were created using the SUPERVISELY polygon tool (supervisely.com). Polygons mark segment annotations by placing waypoints along the boundaries of the objects that the model must segment. All annotations were reviewed by three nephrologists with extensive experience in nephropathology. The pathologists engaged in discussions to resolve disagreements. Four predefined classes were annotated: (1) glomerulus, (2) healthy tubules, (3) necrotic tubules, and (4) tubules with casts. Figure 1A, B and C show examples of the whole-slide images of H&E and PAS-stained kidney section obtained using a slide scanner and a randomly generated patch without annotations, respectively. The annotations consisting of four different structures, ‘glomerulus,’ ‘healthy tubules,’ ‘necrotic tubules,’ and ‘tubules with cast’ are shown in Fig. 1D. In total, 27,478 annotations, along with their corresponding patches, were partitioned into two distinct proportions: a training subset comprising 80% of the data and a testing subset constituting the remaining 20%. Patches that belonged to the same WSI did not appear in either the training or testing proportions to ensure robust generalization of the segmentation models. Subsequently, to fine-tune the model hyperparameters, the training subset underwent further random splitting into training (80%) and validation (20%) subsets. This approach aimed to facilitate the refinement of model performance by iteratively adjusting the hyperparameters based on the validation set, while preserving the independence of the testing set for the final evaluation of model generalization (Table 1 and Figs. 2, 3 and 4).

**Table 1:** The number of annotations in each class used in training and test set for segmentation model.

Class	Training set (Total = 22,951)	Test set (Total = 4527)
Glomerulus	731	141
Healthy tubules	11,915	2249
Necrotic tubules	7362	1684
Tubules with cast	2943	453

### Preprocessing

Because the pathology images were represented in an RGB data structure, the pixel values of the images ranged from 0 to 255. The pixels were scaled to a range between zero and one to avoid gradient explosions during the training phase. The patch images were resized to 512 × 512 pixels before being fed into the deep-learning model for segmentation. Three different augmentation methods were used to address overfitting resulting from a limited number of samples: horizontal flipping, rotation, and brightness adjustment. The third augmentation method was used because of varying degrees of slide brightness. Although we performed PAS staining for all histological slides using the same protocol, the degree of staining and, consequently, the overall brightness of the specimen may have differed among the different slides because the tissue embedded in paraffin was collected at various times. Thus, a random adjustment of the contrast of patches can improve model performance. The augmentation methods were applied only to the training and validation datasets, and not to the test set. All augmentation protocols were implemented using the Python Albumentation library^15^. We applied 3 augmentation methods to the 50% of the training images: (1) horizontal flip, (2) rotate images with random angles from − 90 to 90°, and (3) contrast change.

### Proposed model framework

In this study, we proposed to use DeepLabV3^16^, which is a two-stage segmentation framework for the segmentation task. The architecture of the DeepLabV3 encoder consists of Atrous Spatial Pyramid Pooling (ASPP) blocks that allow it to maintain the Field-of-View (FOV) of the network layers and effectively capture contextual information at different scales. Moreover, DeepLabV3 uses dilated or “-atrous” convolution layers to maintain high-precision predictions while maintaining a wide FOV. This is particularly critical for histopathological imaging because of the fine-grained structures and textures. In addition, the dense structure of the images leads to an extreme foreground–background class-imbalance phenomenon. To overcome this challenge, we integrated an objective function, which is the summation of the Dice Loss^17^ and Focal Loss^18^ functions. Unlike classification tasks, the outputs of segmentation problems are continuous, rather than categorical. Thus, Dice Loss is particularly suitable for continuous maps because it measures the overlap between a prediction and target. Furthermore, Dice Loss is independent of the statistical distribution of labels and penalizes misclassifications based on the overlap between the predicted regions and ground truths. The last part of our object function is the Focal Loss function, which was used in the RetinaNet^18^ deep-learning model to mitigate the class-imbalance problem in dense object detection. Furthermore, we integrated DeepLabV3 with a MobileNet backbone designed for mobile and embedded devices such that the developed model can be applied to devices that might have limited computational resources in clinical environments.

As presented in Table 2, our datasets were imbalanced, with the number of annotations for the Glomerulus class being relatively small compared to the other classes. To address this issue, the objective function assigns a higher weight to examples in the minority class, and a lower weight to those in the majority class. Mathematically, the objective function can be described by the following equation:

1 $$L(y,\overline{p}) = 1 - \frac{{\left( {2y\overline{p} + 1} \right)}}{{\left( {y + \overline{p + 1} } \right)}} - \left( {y - \overline{p}} \right)^{\gamma } \log_{b} \left( {\overline{p}} \right),$$

where $y$, $\overline{p }$, and $\gamma$ correspond to the ground truth, model prediction, and the parameter that controls the degree of focus on the difficulty of the examples, respectively. If $\gamma$ is set to 0, the Focal Loss is reduced to the standard cross-entropy loss. The proposed model was implemented using PyTorch^19^, and the loss function was obtained from the MONAI library^19,20^. The training procedure took approximately 4 h on a graphics processing unit (GPU) RTX 3090 24 GB.

**Table 2:** Quantitative segmentation performance of four classes in the actue tublar injury images in training, validation and testing sets.

	DICE (%)			
	Glomerulus	Healthy tubules	Necrotic tubules	Tubules and cast
Training	96.19 $\pm$ 2.96	94.77 $\pm$ 1.62	90.89 $\pm$ 22.14	96.49 $\pm$ 3.41
Validation	95.91 $\pm$ 13.04	94.76 $\pm$ 2.79	90.09 + 22.05	96.38 $\pm$ 2.81
Testing	91.78 $\pm$ 11.09	87.37 $\pm$ 4.02	88.08 $\pm$ 6.83	83.64 $\pm$ 20.39

	IoU (%)			
	Glomerulus	Healthy tubules	Necrotic tubules	Tubules and cast
Training	93.19 $\pm$ 4.89	90.12 $\pm$ 2.79	87.64 $\pm$ 22.19	93.42 $\pm$ 6.07
Validation	93.06 $\pm$ 13.04	90.15 $\pm$ 4.42	86.59 $\pm$ 21.94	93.18 $\pm$ 5.23
Testing	86.09 $\pm$ 12.87	77.79 $\pm$ 6.11	79.36 $\pm$ 10.89	75.49 $\pm$ 21.21

	Sensitivity (%)			
	Glomerulus	Healthy tubules	Necrotic tubules	Tubules and cast
Training	86.29 ± 28.77	94.96 ± 1.97	65.78 ± 43.85	62.45 ± 45.27
Validation	80.23 ± 35.39	94.96 ± 1.66	67.03 ± 43.74	64.54% ± 44.77
Testing	84.84 ± 27.11	86.72 ± 6.49	75.96 ± 32.59	69.44% ± 35.87

	Specificity (%)			
	Glomerulus	Healthy tubules	Necrotic tubules	Tubules and cast
Training	99.87 ± 1.23	91.72 ± 9.29	97.03 ± 3.06	99.26 ± 1.97
Validation	99.89 ± 1.34	91.33 ± 10.54	96.89 ± 2.61	99.25 ± 0.89
Testing	99.69 ± 2.65	90.25 ± 10.04	88.54 ± 7.32	98.74 ± 1.29

	Accuracy (%)			
	Glomerulus	Healthy tubules	Necrotic tubules	Tubules and cast
Training	99.69 ± 0.03	95.39 ± 0.28	97.13 ± 0.24	98.97 ± 1.15
Validation	99.72 ± 0.11	91.32 ± 2.85	96.89 ± 2.67	98.95 ± 1.15
Testing	99.43 ± 0.37	90.95 ± 3.27	90.07 ± 5.13	97.98 ± 1.85

### Data analyses

Network performance was quantitatively assessed using instance-level DICE and IoU scores. In image segmentation, the DICE and IoU are commonly used to evaluate the performance of segmentation algorithms. They measured the similarity between the predicted segmentation mask and ground-truth mask. While DICE measures the ratio of the intersection of the two masks to the sum of their areas, the IoU metric calculates the overlap between the predictions and human masks by taking the ratio of their intersection to their union. In addition, sensitivity, specificity, and accuracy were calculated. In this study, we used these metrics to evaluate the performance of the proposed system comprehensively.

### Comparison with other model

In our comprehensive comparative analysis, we used U-Net^21^ and SegFormer^22^, two widely used neural network architectures. U-Net, a widely used convolutional neural network architecture for semantic segmentation, features a distinctive U-shaped design comprising the contracting, bottleneck, and expansive paths. It excels at capturing intricate spatial features and is known for its success in medical image segmentation tasks. SegFormer, a state-of-the-art algorithm for segmentation, adopts a transformer-based architecture^23^ with lightweight multilayer perception. It demonstrates an extremely high level of performance on the Cityscapes^24^ dataset, highlighting its effectiveness in diverse computer vision applications. We applied the standard architectures of U-Net and SegFormer without modification and used the same training, validation, and test subsets as in our model. The DICE and IoU values of U-Net and SegFormer were measured for comparison.

### Statistical analyses

We used One-way ANOVA (or t-tests) for comparison between deepLabV3, UNet and Segformer by comparing respective Dice and IoU coeffecienct. *P* < 0.05 was considered statistically significant.

## Results

### Model parameter optimization

We trained the model using the following hyperparameters: a learning rate of 0.5, batch size of 32, 60 epochs, and $\gamma$ of 2. We evaluated the performance of each combination of hyperparameters using a held-out validation dataset. We found that the learning rate had a significant impact on model performance, with higher learning rates leading to faster convergence but a lower Dice coefficient (DICE) and Intersection over Union (IoU). In contrast, a lower learning rate results in overfitting. The batch size had a less pronounced effect, with a larger batch size generally resulting in faster convergence and improved validation performance. In addition to learning rate and batch size, we discovered that $\gamma$ of Focal Loss was very sensitive to the performance of the model. A small value led to overfitting of the majority classes, whereas a large value resulted in poor performance in the training dataset.

### Performance of segmentation model

The effectiveness of the proposed segmentation model for each class is summarized in Table 2. The average (± standard deviation) DICE scores for the glomerulus, healthy tubules, necrotic tubules, and tubules with cast were 91.78 ± 11.09, 87.37 ± 4.02, 88.08 ± 6.83, and 83.64 ± 20.39%, respectively. These results suggest that the proposed segmentation model is highly accurate in identifying different classes of objects, with the glomerulus class achieving the highest DICE score. Analysis of the IoU scores yielded similar results. The average (± standard deviation) IoU for the glomerulus, healthy tubules, necrotic tubules, and tubules with cast were 86.09 ± 12.87, 77.79 ± 6.11, 79.36 ± 10.89, and 75.49 ± 21.21%, respectively, thus demonstrating the accuracy of the proposed segmentation model across all classes with the glomerulus class achieving the highest IoU score.

In addition, the sensitivity, specificity, and accuracy of the proposed model were evaluated. The sensitivity values for the glomerulus, healthy tubules, necrotic tubules, and tubules with cast were 84.84 ± 27.11, 86.72 ± 6.49, 75.96 ± 32.59, and 69.44 ± 35.87%, respectively. The specificity values for the glomerulus, healthy tubules, necrotic tubules, and tubules with cast were 99.69 ± 2.65, 90.25 ± 10.04, 88.54 ± 7.32, and 98.74 ± 1.29%, respectively. The accuracy values for the glomerulus, healthy tubules, necrotic tubules, and tubules with cast were 99.43 ± 0.37, 90.95 ± 3.27, 90.07 ± 5.13, and 97.98 ± 1.85%, respectively.

### Comparison with other studies

We compared our model with existing state-of-the-art methods (U-Net and SegFormer) for histopathological assessment of renal tubular injury. Table 3 presents a comparison between the performances of the three models for the testing subset. Our model (DeepLabV3) exhibited a comparable or slightly better performance than SegFormer. The performance of the proposed model was better than that of U-Net, particularly in segmenting necrotic tubules and tubules with cast.

**Table 3:** Comparison of testing performance between our model (DeepLabV3), Segformer, and U-Net.

	DICE (%)			
	Glomerulus	Healthy tubules	Necrotic tubules	Tubules and cast
DeepLabV3	91.78 $\pm$ 11.09	87.37 $\pm$ 4.02	88.08 $\pm$ 6.83	83.64 $\pm$ 20.39
Segformer	84.39 ± 24.94	86.69 ± 0.44	75.69 ± 26.95	79.19 ± 24.88
U-Net	80.44 ± 24.64	82.18 ± 5.90	64.81 ± 28.81	53.66 ± 30.17

	IoU (%)			
	Glomerulus	Healthy tubules	Necrotic tubules	Tubules and cast
DeepLabV3	86.09 $\pm$ 12.87	77.79 $\pm$ 6.11	79.36 $\pm$ 10.89	75.49 $\pm$ 21.21
Segformer	76.76 ± 26.51	76.77 ± 0.69	61.55 ± 27.12	64.81 ± 28.81
U-Net	72.78 ± 23.73	70.08 ± 8.36	53.41 ± 24.59	53.66 ± 30.17

## Discussion

Over the last decade, numerous studies have focused on the development of deep-learning models for nephropathology. In several previous studies, neural networks have been trained and successfully applied to specific glomerular segmentation tasks, such as distinguishing between glomerular and non-glomerular regions and classifying healthy and injured glomeruli in WSIs of both human disease and animal models^25–27^. In 2020, Uchino et al. developed a comprehensive deep-learning model to classify multiple glomerular images and suggested its potential use in enhancing the diagnostic accuracy for clinicians^28^.

The initial results of the multiclass segmentation task for kidneys were reported in 2018^29^. They proposed a method for renal segmentation of PAS-stained digital slides of renal allograft resections using CNNs for nine classes, including five healthy structures (glomerulus, distal tubules, proximal tubules, arterioles, and capillaries) and four pathological structures (atrophic tubules, sclerotic glomeruli, fibrotic tissue, and inflammatory infiltrates). Three different network architectures were used to perform this task: a fully convolutional network, U-net, and a multiscale fully convolutional network.

Another CNN for the multiclass segmentation of kidney sections with PAS staining was developed by Hermsen et al.^30^. Dice coefficients were used to assess the segmentation performance for ten classes (glomerulus, sclerotic glomerulus, empty Bowman's capsules, proximal tubules, distal tubules, atrophic tubules, undefined tubules, arteries, interstitium, and capsule) of nephrectomy and transplant biopsy specimens. In both datasets, the glomerulus was the best-segmented class (Dice coefficients of 0.95 and 0.94)^30^. Recently, Bouteldja et al. published high-performance deep-learning algorithms for the multiclass segmentation of kidney histology for various diseases in mouse models and other species. In this study, six annotated structures were used: tubules, full glomerulus, glomerular tuft, artery, arterial lumen, and vein^31^. Although previous studies have focused on developing models for segmenting renal tubular structures, the predefined classes of tubules included only normal tubular types, such as proximal and distal tubules, or abnormal tubular types, such as atrophic tubular structures, in a renal fibrosis model^32^.

To the best of our knowledge, there have been a limited number of reports on segmentation models for identifying injured tubules in patients with acute kidney injury. Our study presents a deep learning-based segmentation model for evaluating acute renal tubular injury in digitized PAS-stained images. We applied deep-learning models to identify the typical structural types of toxicity-induced acute tubular injuries, including glomeruli, healthy tubules, necrotic tubules, and tubules with casts. The DICE scores and IoU showed high and consistent performances in the segmentation of these regions. Notably, the performance of the proposed model was the highest for the glomerulus despite the glomerulus class having the smallest number of annotations. This suggests that the performance of the model can be improved further by adding more training data, particularly for the glomerulus class. Overall, the results suggest that the proposed segmentation model has the potential to be used in clinical applications for the accurate identification and segmentation of different kidney structures, particularly injured tubules. In future, we intend to translate the technique developed in this study to a human biopsy dataset. As a dissociation exists between histopathological findings and the clinical symptoms of AKI in some cases (such as volume depletion-induced AKI in allergic, cardiogenic, or hemorrhagic shock), renal biopsy may assist in assessing structural injury, differentiating the cause of AKI, and aiding in treatment^1^.

The proposed approach exhibited a similar or slightly higher performance than the state-of-the-art models. The mean DICE values for SegFormer and U-Net were 81.49% (ranging from 75.69 to 86.69%) and 70.27% (ranging from 53.66 to 82.18%), respectively, across the four classes, whereas our model yielded a mean DICE of 87.71% (ranging from 83.64 to 91.78%). The mean IoUs for SegFormer and U-Net were 69.97% (ranging from 61.55 to 76.77%) and 62.48% (ranging from 53.41 to 72.78%) across the four classes, respectively, whereas our model had a mean IoU of 79.68% (ranging from 75.49 to 86.09%). Therefore, compared with previously used methods for assessing renal tubular injury, the method proposed in this study may be effective for identifying injured renal tubules in acute kidney injury in terms of segmentation performance and computational complexity. It is noteworthy that our model exhibited a comparable or slightly better performance than Segformer, with significantly simpler computational complexity. SegFormer produced results with a high degree of parameter counts of 64 million, whereas our model, DeepLabV3, based on Mobile-net, presented relatively high efficiency with only 11 million parameter counts. This efficiency underscores the potential practical advantages of our model in terms of computational resources and model complexity.

Our study has some limitations. First, a deep-learning model was developed to evaluate the histological images of murine cisplatin-induced acute tubular injury. Although the histological structures of the mouse and human kidneys are similar, the distance or connective tissue area among the structures in the mouse kidney tissue is relatively small compared to that in humans. These closely located structures make it more difficult to distinguish the boundaries between them, particularly in necrotic areas where the basement membranes are occasionally not intact. Second, the number of WSIs and patches generated in this study was limited. A study that includes a larger number of annotations is underway and is expected to achieve higher performance in training the model. Third, when substances such as casts are present in the injured tubular lumen, the effectiveness of measuring the degree of tubular injury decreases.

## Conclusion

The deep-learning segmentation model developed in this study can accurately identify the histopathological structures of injured renal tubules. The results serve as the basis for future studies with larger datasets, including mouse and human biopsy samples, which can provide new opportunities for applying the proposed methods to renal pathology.

## Data Availability

The datasets used and/or analysed during the current study available from the corresponding author on reasonable request.

## References

[1] Gaut JP, Liapis H (2021). Acute kidney injury pathology and pathophysiology: a retrospective review. Clin. Kidney J..

[2] Tavares MB (2012). Acute tubular necrosis and renal failure in patients with glomerular disease. Ren. Fail..

[3] Racusen LC (1995). The histopathology of acute renal failure. New Horiz..

[4] Khwaja A (2012). KDIGO clinical practice guidelines for acute kidney injury. Nephron Clin. Pract..

[5] Okusa MD, Davenport A (2014). Reading between the (guide)lines–the KDIGO practice guideline on acute kidney injury in the individual patient. Kidney Int..

[6] Ramesh AN, Kambhampati C, Monson JR, Drew PJ (2004). Artificial intelligence in medicine. Ann. R. Coll. Surg. Engl..

[7] Miotto R, Wang F, Wang S, Jiang X, Dudley JT (2018). Deep learning for healthcare: Review, opportunities and challenges. Brief Bioinform..

[8] Park K (2023). Deep learning predicts the differentiation of kidney organoids derived from human induced pluripotent stem cells. Kidney Res. Clin. Pract..

[9] Becker JU (2020). Artificial intelligence and machine learning in nephropathology. Kidney Int..

[10] Ghaznavi F, Evans A, Madabhushi A, Feldman M (2013). Digital imaging in pathology: whole-slide imaging and beyond. Annu. Rev. Pathol..

[11] Huo Y, Deng R, Liu Q, Fogo AB, Yang H (2021). AI applications in renal pathology. Kidney Int..

[12] Salvi M (2021). Automated assessment of glomerulosclerosis and tubular atrophy using deep learning. Comput. Med. Imaging Graph..

[13] Jung YJ, Park W, Kang KP, Kim W (2020). SIRT2 is involved in cisplatin-induced acute kidney injury through regulation of mitogen-activated protein kinase phosphatase-1. Nephrol. Dial. Transplant..

[14] Jiang L (2021). A deep learning-based approach for glomeruli instance segmentation from multistained renal biopsy pathologic images. Am. J. Pathol..

[15] Buslaev, A. *et al.* Albumentations: fast and flexible image augmentations. arXiv:1809.06839 (2018).

[16] Chen, L. C., Papandreou, G., Schroff, F. & Adam, H. Rethinking atrous convolution for semantic image segmentation. arXiv:1706.05587 (2017).

[17] Sudre CH, Li W, Vercauteren T, Ourselin S, Jorge Cardoso M (2017). Generalised dice overlap as a deep learning loss function for highly unbalanced segmentations. Deep Learning in Medical Image Analysis and Multimodal Learning for Clinical Decision Support.

[18] Lin TY, Goyal P, Girshick R, He K, Dollar P (2020). Focal loss for dense object detection. IEEE Trans. Pattern Anal. Mach. Intell..

[19] Paszke, A. *et al.* Pytorch: An imperative style, high-performance deep learning library. arXiv:1912.01703 (2019)

[20] Cardoso, M. J. *et al.* MONAI: An open-source framework for deep learning in healthcare (2022).

[21] Ronneberger, O., Fischer, P. & Brox, T. U-Net: Convolutional Networks for Biomedical Image Segmentation. *CoRR* https://arxiv.org/abs/1505.04597 (2015).

[22] Xie, E., Wang, W., Yu, Z., Anandkumar, A., Alvarez J. M. & Luo, P. SegFormer: Simple and efficient design for semantic segmentation with transformers. In *Advances in Neural Information Processing Systems*. 10.48550/arXiv.2105.15203 (2021).

[23] Vaswani, A., Shazeer, N., Parmar, N., Uszkoreit, J., Jones, L., Gomez, A. N., Kaiser, Ł. & Polosukhin, I. Attention is all you need. In *Advances in Neural Information Processing Systems* (eds. Guyon, I., Von Luxburg, U., Bengio, S., Wallach, H., Fergus, R., Vishwanathan, S. & Garnett, R.) Vol. 30 (Curran Associates, Inc., 2017).

[24] Cordts, M., Omran, M., Ramos, S., Rehfeld, T., Enzweiler, M., Benenson, R., Franke, U., Roth, S. & Schiele, B. The cityscapes dataset for semantic urban scene understanding. In *Proceedings of the IEEE Conference on Computer Vision and Pattern Recognition (CVPR)* (2016).

[25] Kannan S (2019). Segmentation of glomeruli within trichrome images using deep learning. Kidney Int. Rep..

[26] Gadermayr M (2017). Segmenting renal whole slide images virtually without training data. Comput. Biol. Med..

[27] Bueno G, Fernandez-Carrobles MM, Gonzalez-Lopez L, Deniz O (2020). Glomerulosclerosis identification in whole slide images using semantic segmentation. Comput. Methods Programs Biomed..

[28] Uchino E (2020). Classification of glomerular pathological findings using deep learning and nephrologist-AI collective intelligence approach. Int. J. Med. Inform..

[29] de Bel, T. *et al.* Automatic segmentation of histopathological slides of renal tissue using deep learning. In *SPIE*. 10.1117/12.2293717 (2018).

[30] Hermsen M (2019). Deep learning-based histopathologic assessment of kidney tissue. J. Am. Soc. Nephrol..

[31] Bouteldja N (2021). Deep learning-based segmentation and quantification in experimental kidney histopathology. J. Am. Soc. Nephrol..

[32] Ginley B (2019). Computational segmentation and classification of diabetic glomerulosclerosis. J. Am. Soc. Nephrol..

